# Fallopian tube nontuberculous mycobacterial infection caused by *Mycobacterium abscessus* mimicking fallopian tube carcinoma: A case report

**DOI:** 10.1016/j.crwh.2026.e00843

**Published:** 2026-07-21

**Authors:** Chihiro Fukunaga, Shunki Kiyokawa, Kanako Nakamura, Yuka Kaneko, Tadanori Kanazawa, Yuka Manabe, Yuya Nogami, Shunsuke Uno, Yoshifumi Uwamino, Ho Namkoong, Fumiko Yagi, Hiroki Muramoto, Akihisa Ueno, Shigeki Sekine, Kenta Masuda, Wataru Yamagami

**Affiliations:** a Department of Obstetrics and Gynecology, Keio University School of Medicine, Tokyo, Japan; b Faculty of Nursing and Medical Care, Keio University, Kanagawa, Japan; c Department of Laboratory Medicine, Keio University School of Medicine, Tokyo, Japan; d Department of Infectious Diseases, Keio University School of Medicine, Tokyo, Japan; e Department of Radiology, Keio University School of Medicine, Tokyo, Japan; f Division of Diagnostic pathology, Keio University School of Medicine, Tokyo, Japan

**Keywords:** Fallopian tube carcinoma, Nontuberculous mycobacteria, *Mycobacterium abscessus*, Granuloma, Frozen section, Differential diagnosis

## Abstract

Fallopian tube carcinoma is frequently detected at an advanced stage, and its imaging findings and tumor marker profiles are often indistinguishable from those of ovarian or peritoneal carcinoma. Nontuberculous mycobacteria (NTM) are environmental acid-fast bacilli with increasing clinical significance in both immunocompromised and immunocompetent individuals. Infection of gynecologic organs is extremely rare. This report describes a case of fallopian tube NTM infection caused by *Mycobacterium abscessus* that mimicked fallopian tube carcinoma. An 87-year-old woman underwent fluorodeoxyglucose positron emission tomography/computed tomography (FDG-PET/CT) during routine surveillance, which revealed focal FDG uptake in the right adnexal region. Serum CA125 was elevated, and magnetic resonance imaging demonstrated findings suspicious for fallopian tube carcinoma. Primary debulking surgery was performed. Intraoperative frozen-section examination of the right adnexa revealed necrotizing granulomas without evidence of malignancy, raising suspicion of mycobacterial infection. Fresh specimens were submitted for microbiological evaluation. Culture identified *Mycobacterium abscessus*, establishing the diagnosis of fallopian tube NTM infection. The patient was asymptomatic and had no abscess formation. Complete surgical resection was achieved, and no antimicrobial therapy was administered. The patient remained recurrence-free at 1-year follow-up. This case highlights the difficulty of differentiating infectious granulomatous disease from malignancy based on imaging findings alone. Intraoperative frozen-section examination played a crucial role in prompting microbiological investigation and establishing the correct diagnosis.

## Highlights

• Fallopian tube NTM infection mimicked tubal carcinoma on MRI and FGD-PET/CT.
• Serum CA125 elevation further supported suspected malignancy.
• Frozen-section revealed necrotizing granulomas intraoperatively.
• Culture identified *Mycobacterium abscessus*.
• Extrapulmonary NTM disease should be considered in adnexal mass differential.

## 1 Introduction

Primary fallopian tube carcinoma (PFTC) is a rare gynecologic malignancy, and its preoperative diagnosis remains challenging because its clinical presentation and imaging findings often overlap with those of benign inflammatory diseases and other pelvic malignancies [1], [2], [3]. Nontuberculous mycobacteria (NTM) are environmental acid-fast bacilli that increasingly cause extrapulmonary infections [4], [5], [6]. *Mycobacterium abscessus* is a rapidly growing NTM characterized by intrinsic antimicrobial resistance [4]. Tuberculosis remains the most common mycobacterial infection of the female genital tract, whereas nontuberculous mycobacterial infections are exceedingly rare at this site [7]. Therefore, the optimal management of gynecologic NTM infection remains unclear. This report describes a rare case of fallopian tube infection caused by *M. abscessus* that required differentiation from fallopian tube carcinoma.

## 2 Case Presentation

An 87-year-old nulligravid woman underwent annual FDG-PET/CT as part of a routine health surveillance programme, which incidentally revealed focal FDG uptake in the right adnexal region. Her serum CA125 level was elevated to 399 U/mL. She was referred for further evaluation of suspected gynecologic malignancy. She had undergone curative surgery and adjuvant chemotherapy for rectal cancer more than 27 years earlier and had no prior history of tuberculosis or *Mycobacterium avium* complex (MAC) infection.

At presentation, the patient was asymptomatic and afebrile, with stable vital signs. Pelvic examination revealed a mobile uterus approximately 7 cm in size. Pelvic organ prolapse corresponding to Pelvic Organ Prolapse Quantification (POP-Q) stage II and a rectocele were noted. No adnexal masses or tenderness were detected. Transvaginal ultrasonography demonstrated a thin endometrium and a 3-cm intramural leiomyoma. The adnexa were poorly visualized, and no ascites was present. Cervical cytology was negative for intraepithelial lesion or malignancy. Endometrial cytology, which is often performed in Japan when ovarian or fallopian tube malignancy is suspected because malignant cells may exfoliate into the uterine cavity, was suspicious [8]. However, endometrial biopsy showed no evidence of malignancy. Laboratory studies revealed CEA 2.3 ng/mL and CA19–9 10 U/mL (within normal ranges), whereas CA125 remained elevated at 233 U/mL.

**Fig. 1:**
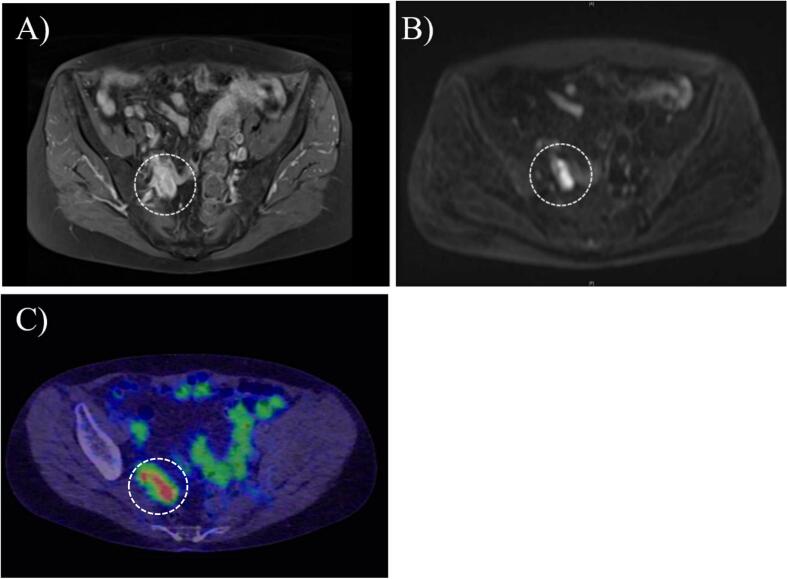
(A) Pelvic magnetic resonance imaging (MRI). Gadolinium-enhanced T1-weighted image. A contrast-enhancing tubular mass is observed in the right adnexal region. The lesion is outlined. (B) MRI. Diffusion-weighted image (DWI). The tubular mass exhibits high signal intensity on DWI. The lesion is outlined. (C) FDG-PET/CT showing increased FDG uptake (SUVmax 9.81) in the right adnexa.

Contrast-enhanced pelvic magnetic resonance imaging (MRI) revealed a band-like lesion in the right adnexal region, raising suspicion of primary fallopian tube carcinoma. The lesion demonstrated intermediate signal intensity on T2-weighted imaging, and diffusion-weighted imaging revealed a band-like hyperintense lesion in the right fallopian tube with abnormal enhancement [Fig. 1A, B]. No lymphadenopathy or distant metastases were identified on the CT scan. FDG uptake (SUVmax 9.81) was observed in the lesion on FDG- PET/CT [Fig. 1C].

**Fig. 2:**
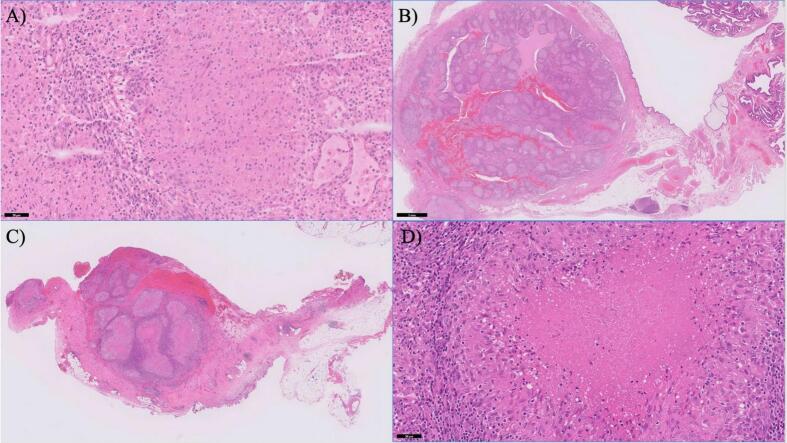
Histopathological findings of the pelvic peritoneum (hematoxylin and eosin staining) and the right fallopian tube (hematoxylin and eosin staining). (A) Frozen-section examination of the right fallopian tube revealed granulomas. (B) Low-power view showing multiple granulomas with necrosis in the right fallopian tube. (C) Low-power view showing similar multiple granulomas with necrosis in the peritoneum of the Douglas pouch. (D) High-power view of the granulomas with necrosis in the peritoneum of the Douglas pouch.

Based on these findings, primary debulking surgery was planned with a preoperative diagnosis of suspected right fallopian tube carcinoma. Intraoperatively, a firm mass was palpated within the right fallopian tube, and two small nodules were scattered over the pelvic peritoneum. The uterus and left adnexa appeared grossly normal. No enlarged pelvic and para-aortic lymph nodes or distant metastases were observed. The right adnexa was submitted for intraoperative frozen-section analysis, which revealed granulomas without evidence of malignancy [Fig. 2A].

Given the preoperative finding of uterine prolapse, total abdominal hysterectomy, bilateral salpingo-oophorectomy, resection of all peritoneal lesions, and posterior colporrhaphy were performed. Based on the frozen-section findings, fresh specimens of the right fallopian tube and ascitic fluid were submitted for acid-fast bacilli staining, tuberculosis polymerase chain reaction (TB-PCR), *mycobacterium avium*-intracellular complex polymerase chain reaction (MAC-PCR), and mycobacterial culture to evaluate for tuberculosis or nontuberculous mycobacterial (NTM) infection.

Permanent histopathological examination of the entire right fallopian tube demonstrated numerous necrotizing granulomas predominantly involving the lamina propria and extending into the muscular layer, with focal involvement of the peritubal stromal tissue [Fig. 2B]. Occasional multinucleated giant cells were identified. No evidence of serous tubal intraepithelial carcinoma (STIC) or occult invasive carcinoma was observed. Similar necrotizing granulomas with chronic inflammatory infiltrates were identified in the peritoneum of the Douglas pouch [Fig. 2C, D]. Ziehl–Neelsen and Grocott staining were negative for acid-fast bacilli and fungi, respectively.

Acid-fast bacilli testing of the right fallopian tube and ascitic fluid, including fluorescent staining, *Mycobacterium tuberculosis* PCR, and MAC-PCR, yielded negative results. However, mycobacterial culture of the right fallopian tube specimen identified *Mycobacterium abscessus* on day 6 after inoculation. Subspecies identification of *Mycobacterium abscessus* was performed using the DNA chromatography method with the KANEKA DNA Chromatography MABC/erm(41) Genotyping Kit, which identified the isolate as *M. abscessus* subsp. *massiliense* [9]*.* Antimicrobial susceptibility testing demonstrated resistance to several antimicrobial agents, including tobramycin and meropenem and so on; however, the isolate remained susceptible to the macrolide antibiotics azithromycin and clarithromycin. In contrast, mycobacterial culture of ascitic fluid remained negative. These findings established the diagnosis of fallopian tube NTM infection caused by *M. abscessus*.

As the patient remained asymptomatic and complete surgical resection had been achieved, postoperative antimicrobial therapy was not administered. The patient remained free of recurrence at 1-year follow-up.

## 3 Discussion

Fallopian tube carcinoma is frequently detected at an advanced stage, and its radiological and tumor marker profiles are often indistinguishable from those of ovarian or peritoneal carcinoma [2]. Although CA125 is widely used as a tumor marker for epithelial ovarian, fallopian tube, and primary peritoneal cancers, it is nonspecific and may also be elevated in benign inflammatory conditions, including pelvic inflammatory disease and granulomatous inflammation [10], [11], [12]. On MRI, fallopian tube carcinoma typically presents as a tubular or sausage-shaped mass with homogeneous signal intensity, low signal intensity on T1-weighted images, high signal intensity on T2-weighted images, and mild-to-moderate contrast enhancement. However, MRI findings of fallopian tube carcinoma vary and frequently overlap with those of benign inflammatory conditions, tubo-ovarian abscesses, endometriosis, and metastatic tumors. Therefore, a definitive diagnosis is often not established until surgical exploration and histopathological examination are performed [3].

The rarity of this disease also contributed to the diagnostic difficulty. Although gynecologic NTM infections have been reported, including infections involving the uterine cervix and uterus [13], [14], [15], no cases of fallopian tube infection caused by NTM have previously been reported, to the authors' knowledge. A PubMed search performed in January 2026 using the terms (“*Mycobacterium abscessus*” OR “nontuberculous mycobacteria”) AND (“fallopian tube” OR “salpinx” OR “salpingitis”) identified no previously reported cases of fallopian tube infection caused by NTM.

Nontuberculous mycobacteria (NTM) comprise more than 200 species of acid-fast bacilli other than *Mycobacterium tuberculosis* and *Mycobacterium leprae*, and are widely distributed environmental organisms [4]. The route of infection in the present case remains uncertain, although one possibility is retrograde ascending infection through the genital tract. The patient reported that she had lived for about six months in a severely unsanitary house while helping to clean the residence of a relative approximately a half year before undergoing FDG-PET/CT. Because NTM are commonly found in water and soil, this exposure may have represented a source of infection. Although this hypothesis remains speculative, retrograde ascending infection through the lower genital tract represents a plausible mechanism in the absence of pulmonary disease or other identifiable infectious foci.

*Mycobacterium abscessus* is one of the major causative organisms of extrapulmonary nontuberculous mycobacterial disease (NTM-EPD), and is known to cause severe infections of the skin and soft tissues. It is regarded as one of the most antibiotic-resistant mycobacterial species. Three subspecies have been identified within the *M. abscessus* complex: *M. abscessus* subsp. *abscessus*, *M. abscessus* subsp. *bolletii*, and *M. abscessus* subsp. *massiliense*, each of which exhibits distinct antimicrobial susceptibility profiles. *M. abscessus* subsp. *abscessus* is known to frequently demonstrate inducible macrolide resistance, resulting in poor therapeutic responsiveness to macrolide-based regimens [4], [6].

No established treatment guidelines exist for NTM-EPD caused by *M. abscessus*. Although multidrug antimicrobial therapy is generally recommended, treatment is often challenging because of its intrinsic antimicrobial resistance [4], [6]. Furthermore, optimal management of gynecologic *M. abscessus* infection remains unclear. In the present case, postoperative antimicrobial therapy was not administered because the patient remained asymptomatic and complete surgical resection had been achieved. The patient remained asymptomatic at 1-year follow-up. However, further accumulation of similar cases is required to determine the optimal management of gynecologic *M. abscessus* infection.

Nine previously reported cases of extrapulmonary NTM infection that required differentiation from malignancy are presented in Table 1 [13], [14], [15], [16], [17], [18], [19]. In all cases, including the present one, malignancy was initially considered the primary differential diagnosis based on imaging, highlighting the difficulty of distinguishing infectious from malignant lesions radiologically. Species identification was achieved in only four cases, all of which involved repeat specimen collection and culture after infection was suspected. These findings highlight the importance of obtaining fresh specimens for mycobacterial culture to establish a definitive diagnosis.

**Table 1:** Previously reported cases of extrapulmonary NTM infection requiring differentiation from malignancy.

Case	Author	Sex	Age	Risk factor	Focus	Symptoms	Initial diagnosis	Operation	Examination	Pathogen	Treatment
1	Ukita M. [13]	F	67	ー	cervix	fatigue and weight loss	cervical cancer	ー	PCR, culture	*M. avium*	antibacterial drugs
2	Tsai TY. [14]	F	41	HIV positive	cervix	irregular bleeding	uterine leiomyosarcoma	+	culture	*M. sherrisii*	antibacterial drugs
3	Gonai Y. [15]	F	78	age	uterine vagina	irregular bleeding	cervical cancer invasion of the vaginal wall	ー	PCR, culture	*M. avium*	antibacterial drugs
4	Zhang L. [16]	M	49	ー	liver	asymptomatic	colorectal cancer metastases	ー	PCR	Unknown	no treatment
5	Zhang L. [16]	F	58	ー	liver	asymptomatic	ovarian cancer metastasis	ー	PCR	Unknown	antibacterial drugs
6	Zhang L. [16]	M	55	ー	liver	asymptomatic	rectal cancer metastases	ー	PCR	Unknown	antibacterial drugs
7	Jung J. [17]	F	64	ー	brain	convulsions	primary unknown brain metastases	+	PCR	Unknown	antibacterial drugs
8	De Piano F. [18]	F	53	ー	peritoneum	asymptomatic	peritoneal cancer	+	staining	Unknown	transferred
9	Sun X. [19]	M	59	immunodeficiency	lung, skin, bones	fever and cough	metastatic malignancy	ー	culture	*M. colombiense*	antibacterial drugs

Diagnostic modalities for NTM infection include acid-fast staining, polymerase chain reaction (PCR), and culture. Among these, culture, although time-consuming, enables the detection of viable organisms and antimicrobial susceptibility testing, both of which are essential for treatment selection. However, formalin-fixed specimens are unsuitable for these analyses, and fresh specimens are required for mycobacterial culture [20]. When tissue sampling is difficult, failure to consider NTM infection preoperatively may result in diagnosis based solely on postoperative pathology. If re-biopsy or additional sampling is not feasible, diagnosis becomes even more difficult. Intraoperative frozen-section examination can provide early evidence suggestive of infection and may facilitate prompt definitive diagnosis and appropriate therapeutic intervention. In the present case, identification of necrotizing granulomas on intraoperative frozen-section examination prompted consideration of mycobacterial infection, leading to submission of fresh tissue for microbiological investigations. Although both Ziehl–Neelsen staining and mycobacterial PCR were negative, culture of the fresh specimen established the diagnosis of *Mycobacterium abscessus* infection. This case highlights that negative Ziehl–Neelsen staining and PCR do not exclude NTM infection, underscoring the importance of submitting fresh tissue for mycobacterial culture whenever granulomatous inflammation raises clinical suspicion of mycobacterial disease.

## 4 Conclusion

This report describes a rare case of fallopian tube NTM infection caused by *Mycobacterium abscessus* that mimicked primary fallopian tube carcinoma. Intraoperative frozen-section examination may serve as a diagnostic fail-safe in infectious diseases that clinically mimic malignancy by prompting consideration of alternative diagnoses. When granulomatous inflammation is identified on frozen-section examination, fresh tissue should be submitted promptly for microbiological culture. This approach facilitates timely definitive diagnosis and enables appropriate selection of antimicrobial therapy.

## Contributors

Chihiro Fukunaga contributed to acquiring and interpreting the data, drafting the manuscript and undertaking the literature review.

Shunki Kiyokawa contributed to acquiring and interpreting the data, drafting the manuscript, undertaking the literature review and revising the article critically for important intellectual content.

Kanako Nakamura, Yuka Kaneko, Tadanori Kanazawa, Yuka Manabe, Yuya Nogami, Kenta Masuda contributed to revising the article critically for important intellectual content from a gynecological perspective.

Shunsuke Uno, Yoshifumi Uwamino, Ho Namkoong contributed to revising the article critically for important intellectual content from an infectious disease perspective.

Fumiko Yagi contributed to acquiring the data, and revised the article critically for important intellectual content from a radiological perspective.

Hiroki Muramoto, Akihisa Ueno, Shigeki Sekine contributed to acquiring the data and revised the article critically for important intellectual content from a histopathological perspective.

Wataru Yamagami contributed to conception of the case report and revised the article critically for important intellectual content.

All authors contributed to patient care. All authors approved the final version of the case report.

## Patient consent

Written informed consent was obtained from the patient for publication of this case report and accompanying images.

## Provenance and peer review

This article was not commissioned and was peer reviewed.

## Declaration of generative AI and AI-assisted technologies in the writing process

During the preparation of this work, the authors used ChatGPT (OpenAI) to improve the English language and readability of the manuscript. The authors reviewed and edited the output as needed and take full responsibility for the content of the published article.

## Funding

No funding from an external source supported the publication of this case report.

## Declaration of competing interest

The authors declare that they have no competing interest regarding the publication of this case report.
